# Longitudinal assessment of the lung mechanics of very low birth weight preterm infants with and without bronchopulmonary dysplasia: Avaliação longitudinal da mecânica pulmonar de crianças pré-termo de muito baixo peso com e sem displasia broncopulmonar

**DOI:** 10.1590/1516-3180.2014.00101812

**Published:** 2015-10-09

**Authors:** Rosane Reis de Mello, Kátia Silveira da Silva, Anniele Medeiros Costa, José Roberto de Moraes Ramos

**Affiliations:** I MD, PhD. Attending Physician, Department of Neonatology, Instituto Nacional de Saúde da Mulher, da Criança e do Adolescente Fernandes Figueira/Fiocruz, Rio de Janeiro (RJ), Brazil.; II MD, PhD. Epidemiologist, Clinical Research Unit, Instituto Nacional de Saúde da Mulher, da Criança e do Adolescente Fernandes Figueira/Fiocruz, Rio de Janeiro (RJ), Brazil.; III MSc. Physiotherapist, Department of Neonatology, Instituto Nacional de Saúde da Mulher, da Criança e do Adolescente Fernandes Figueira/Fiocruz, Rio de Janeiro (RJ), Brazil.; IV MD, PhD. Head of Department of Neonatology, Instituto Nacional de Saúde da Mulher, da Criança e do Adolescente Fernandes Figueira/Fiocruz, Rio de Janeiro (RJ), Brazil.

**Keywords:** Respiratory mechanics, Respiratory function tests, Bronchopulmonary dysplasia, Infant, premature, Lung compliance, Mecânica respiratória, Testes de função respiratória, Displasia broncopulmonar, Prematuro, Complacência pulmonar

## Abstract

**CONTEXT AND OBJECTIVE:**

Prematurity has been correlated with altered lung mechanics. Some infants develop lung injury as a consequence of lung immaturity, invasive mechanical ventilation and exposure to oxygen, thus resulting in bronchopulmonary dysplasia. The aim here was to compare the lung mechanics of preterm infants with and without bronchopulmonary dysplasia during the first year of life.

**DESIGN AND SETTING:**

Prospective cohort study in a tertiary-level hospital.

**METHODS:**

This study included premature infants at a public hospital who underwent two pulmonary function tests: one at discharge and the other at the corrected age of 4 to 8 months. Tidal volume, lung compliance and lung resistance were measured. Statistical tests were used for comparisons between infants with and without bronchopulmonary dysplasia.

**RESULTS:**

102 children with mean gestational age of 29 ± 2.0 weeks were studied; 17 with bronchopulmonary dysplasia. Lung compliance (0.84 ± 0.29 versus 1.28 ± 0.46; P < 0.001) and tidal volume (6.1 ± 0.94 versus 7.2 ± 1.43; P < 0.01) at discharge were significant lower in children with bronchopulmonary dysplasia than in those without the disease, but no differences were observed at the second test (compliance: 1.53 ± 0.77 versus 1.94 ± 1.01; P = 0.12; and tidal volume: 6.9 ± 1.4 versus 7.3 ± 1.6; P = 0.42).

**CONCLUSION:**

Differences in lung mechanics were observed between infants with and without bronchopulmonary dysplasia at hospital discharge but these differences were no longer detected at the final follow-up. The lung mechanics of all the infants improved over this period of time.

## RESUMO

**CONTEXTO E OBJETIVO:**

Prematuridade tem sido associada com mecânica pulmonar alterada. Algumas crianças desenvolvem lesão pulmonar como consequência de imaturidade pulmonar, ventilação mecânica invasiva e exposição a oxigênio, resultando em displasia broncopulmonar. O objetivo foi comparar a mecânica pulmonar de prematuros com e sem displasia broncopulmonar durante o primeiro ano de vida.

**DESENHO DO ESTUDO E LOCAL:**

Estudo de coorte prospectivo em um hospital terciário.

**MÉTODOS:**

O estudo incluiu prematuros, de hospital público, que realizaram duas provas de função pulmonar, uma na alta e outra entre quatro e oito meses de idade corrigida. Foram mensurados o volume corrente, a complacência e a resistência pulmonares. Testes estatísticos foram usados para comparações entre crianças com e sem displasia broncopulmonar.

**RESULTADOS:**

Foram estudadas 102 crianças com idade gestacional média de 29 ± 2 semanas; 17 com displasia broncopulmonar. A complacência pulmonar (0,84 ± 0,29 *versus * 1,28 ± 0,46; P < 0.001) e o volume corrente (6,1 ± 0,94 *versus * 7,2 ± 1,43; P < 0.01) na alta foram significativamente inferiores nas crianças com displasia broncopulmonar comparadas às crianças sem a doença, mas não foram observadas diferenças significativas no segundo teste (complacência: 1.53 ± 0.77 *versus * 1.94 ± 1.01; P = 0.12; e volume corrente: 6.9 ± 1.4 *versus * 7.3 ± 1.6; P = 0.42).

**CONCLUSÃO:**

Diferenças na mecânica pulmonar foram observadas entre crianças com e sem displasia broncopulmonar na alta hospitalar, mas essas diferenças não foram detectadas no seguimento final. A mecânica pulmonar de todas as crianças melhorou no decorrer desse período de tempo.

## INTRODUCTION

Bronchopulmonary dysplasia is a chronic lung disease that mostly affects prematurely born children. The disease is associated with use of mechanical ventilation and prolonged use of oxygen. Although these children are nowadays managed with "lung protective strategies", such as prenatal steroids, exogenous surfactants and minimally invasive ventilation strategies, along with other advances in neonatal care, bronchopulmonary dysplasia remains one of the most common complications of prematurity.^1^ The main characteristic of children with bronchopulmonary dysplasia is late development of the lung acinus with abnormal alveolarization, abnormal deposition of elastin and abnormal vascularization.^2^ These lung alterations may have significant consequences that go beyond childhood, and may result in obstruction and hyperreactivity of the airways.^3^ Infants with "new bronchopulmonary dysplasia" exhibit abnormalities in lung function after birth, throughout childhood and into adolescence.^4^

Moreover, a few studies conducted during the last decade have evaluated the progression or regression of alterations to lung function detected before medical discharge in preterm infants.^5^ Extreme prematurity results in persistent alveolar damage.^6^ Poor lung function during childhood has been reported in both healthy preterm infants and in those with bronchopulmonary dysplasia, although it is unclear whether prematurity alone can explain the reduction in lung function observed in infants with bronchopulmonary dysplasia.^7^ Moreover, it has not yet been established how the lung grows during bronchopulmonary dysplasia and how the immature lung recovers from this disease.^5^

A longitudinal evaluation of lung function after premature infants have been discharged may help to identify those at risk of incomplete recovery of respiratory function and hence of development of respiratory problems during childhood.^5^

### OBJECTIVE

The purpose of this study was to compare the development of lung function at two times during the first year of life of very low birth weight preterm infants with and without bronchopulmonary dysplasia.

## METHODS

### Study design and inclusion criteria

**Figure 1:**
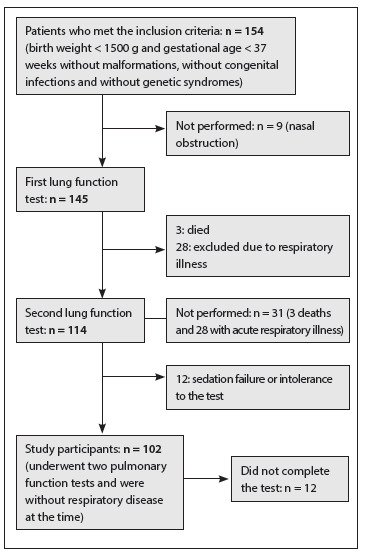
Patient flow chart.

From 2005 to 2009, a prospective cohort study was conducted among very low birth weight premature infants (birth weight < 1500 g) who were admitted to and treated at a public neonatal intensive care unit. The protocol included two pulmonary function tests, performed at the time of discharge from the unit and at the corrected age of 4 to 8 months (Figure 1). Infants with congenital malformations, genetic syndromes or congenital infections and those who did not undergo the two lung function tests were excluded. 

### Evaluation of pulmonary function

Only infants who were clinically stable, were breathing room air and did not show any signs of respiratory infection over the three weeks prior to the tests were studied. Body weight and length were recorded prior to the tests. The pulmonary function tests were done at the lung function laboratory of our institution and were performed by a neonatologist and a physiotherapist who were unaware of theinfants' medical history. Recordings were made with the infant in an incubator during quiet sleep, i.e. non-rapid eye movement (non-REM) sleep. No sedation was used for the first test, but chloral hydrate at a dosage of 50 mg/kg, 15 to 30 minutes beforehand, was given for the second pulmonary function test. The first test was performed during the week scheduled for hospital discharge. 

The pulmonary function evaluation included recordings of the following physiological signs: airflow, esophageal pressure and airways pressure. The PCLAB (Data Translation) software was used for data acquisition. Airflow was measured using a resuscitation-type mask coupled to a pneumotachograph (Fleisch 00). Tidal volume was obtained through electronic integration of the flow signal. Esophageal pressure was measured with a water-filled catheter positioned in the distal third of the esophagus and connected to an ultra-sensitive pressure transducer (Validyne MP45). Airway pressure was measured using an air-filled catheter placed at the resuscitation mask and connected to another pressure transducer (Validyne DP45). 

The recordings were saved into a computer and the Anadat/Labdat software (Infodat, Montreal, Canada) was used for data analysis. Tidal volume, respiratory frequency, lung compliance and resistance were calculated. For each patient, 20 to 50 respiratory cycles were selected in order to calculate pulmonary resistance and compliance, using the linear regression method (computed calculation/Abreath-Anadat). 

Lung compliance and the tidal volume were adjusted by dividing their values by the body weight.8,9 Lung compliance values < 1.2 ml/cm H2O/kg were considered abnormal.9,10 Lung resistance was considered abnormal if it was greater than 50 cm H2O/l/sec.^11^

All infants were clinically monitored through monthly visits to our hospital's outpatient follow-up clinic for at-risk newborns.

### Definitions 

Bronchopulmonary dysplasia was defined as a situation of use of oxygen therapy at 36 weeks of corrected age.^12 ^The classification of the adequacy of the infant's weight for the gestational age was based on Alexander's intrauterine growth curve.13 Infants with birth weight below the 10th percentile were classified as small for the gestational age (SGA) and those with birth weight between the 10th and 90th percentiles were considered to be appropriate for the gestational age (AGA). Septicemia was defined as the presence of a positive blood culture.

### Sample size and statistical analysis

A sample size of 105 children (21 exposed and 84 unexposed) was estimated, taking into account the following parameters: expected frequency of functional impairment of 40% for infants with bronchopulmonary dysplasia (exposed group) and 10% for the unexposed group; relative risk of functional impairment at 4-8 months of 4.0; significance level of 0.05; and power of 80%. 

The data were analyzed by using the Epi Info software (CDC, USA). Appropriate descriptive statistics were used for the categorical variables. The t test was used for variables with normal distribution and Kruskal-Wallis for those without normal distribution. The chi-square or Fisher's test was applied to determine ratio differences. The statistical significance level was set at 5%.

This study was approved by the Research Ethics Committee of Instituto Fernandes Figueira and all the infants' parents signed a consent statement.

## RESULTS 

Over the period between 2005 and 2009, 154 newborns met the inclusion criteria. Close to the time of medical discharge from the neonatal intensive care unit, 145 newborns (94.2%) underwent the pulmonary function test at a gestational age of 38 ± 3 weeks. For 9 patients, the first test was not done because of nasal obstruction, in conjunction with a positive family history of respiratory viral infection. Of the 145 newborns, 3 children died after medical discharge from the neonatal unit, and it was not possible to apply the second test to another 28 due to respiratory disease during the period established for the test. Three of these 28 children had bronchopulmonary dysplasia. 

The second test was thus performed on 114 infants (74%), at an age of 5 ± 2.2 months. Twelve did not complete the second test due to technical problems such as sedation failure and intolerance to the esophageal catheter (Figure 1). No statistically significant differences in birth weight, gestational age, duration of mechanical ventilation, duration of oxygen therapy or frequency of bronchopulmonary dysplasia were observed between the patients studied and those who were excluded.

The sample for this study therefore included 102 premature infants with birth weight under 1500 g, who underwent two pulmonary function tests and did not have any symptoms of respiratory disease. The characteristics of the population studied are described in Table 1. The mean gestational age of the sample was 29 weeks, and more than 80% of the mothers underwent antenatal corticosteroid therapy to improve the lung condition of their infants. Almost 70% of the sample required mechanical ventilation and 95% of the infants who developed bronchopulmonary dysplasia underwent mechanical ventilation. The incidence of male babies was almost twice as high among infants with bronchopulmonary dysplasia as among those without bronchopulmonary dysplasia. 

**Table 1:** Characteristics of the sample of very low birth weight premature infants with and without bronchopulmonary dysplasia

Maternal and neonatal characteristics	Bronchopulmonary dysplasia		P
	Yes (n = 17)	No (n = 85)	
Tobacco exposure during pregnancy	1 (5.8)	8 (9.4)	1.0
Antenatal corticosteroids	14 (82.4)	80 (94.1)	0.24
Birth weight (grams)	1001 [265]	1097 [236]	0.13
Gestational age (weeks)	28 [2]	29 [2]	0.15
Male	14 (82.4)	38 (44.7)	< 0.01
Small for gestational age	7 (41.2)	32 (37.6)	0.78
Mechanical ventilation	16 (94.1)	52 (61.2)	< 0.01
Oxygen therapy (hours)	1916 [1096]	375.3 [465]	< 0.001
Patent ductus arteriosus	10 (58.8)	39 (45.9)	0.32
Intracranial hemorrhage	6 (35.3)	17 (20.0)	0.28
Septicemia	3 (17.6)	9 (10.6)	0.68
Postnatal corticosteroids	4 (23.5)	1 (1.2)	< 0.01

Continuous variables are presented as mean [standard deviation]. Categorical variables are presented as n (percentage).

The mean age at the time of the pulmonary function test, which was performed close to the time of medical discharge, was 37 ± 3.0 weeks for the children with bronchopulmonary dysplasia and 38 ± 3.0 weeks for those without the disease (P = 0.27). The lung compliance was abnormal in 88.2% (n = 15/17) of the children with bronchopulmonary dysplasia and in 48% (41/85) of those without bronchopulmonary dysplasia (P = 0.002). The lung resistance was abnormal in 94% of the children with bronchopulmonary dysplasia and in 80% of those without bronchopulmonary dysplasia. The second lung function test was applied at a mean age of 5.4 ± 2.6 months for the children with bronchopulmonary dysplasia and 4.9 ± 2.1 months for those without the disease.

At 4-8 months of life, the children with bronchopulmonary dysplasia also had a higher rate of change in lung compliance (47%) than did the children without bronchopulmonary dysplasia (11.7%) (P = 0.001). Similar to what was seen at the time of medical discharge, there was no statistical difference in the frequency of impaired lung resistance between the children with bronchopulmonary dysplasia (29.4%) and those without the disease (22.4%). There were reductions in the frequencies of impaired pulmonary compliance and lung resistance, in both groups.

As shown in Table 2, the mean lung compliance close to the time of medical discharge from the neonatal unit among the children with bronchopulmonary dysplasia was considerably lower than the mean lung compliance among the children without bronchopulmonary dysplasia. The mean tidal volume of the children with bronchopulmonary dysplasia was considerably different from that to the children without bronchopulmonary dysplasia. There was no difference in anthropometric measurements in the first test between those with and without bronchopulmonary dysplasia.

**Table 2:** Pulmonary mechanics variables among infants with and without bronchopulmonary dysplasia: mean [standard deviation]

Characteristics	Close to time of discharge		P	At time of follow-up		P
	Bronchopulmonary dysplasia			Bronchopulmonary dysplasia		
	Yes (n = 17)	No (n = 85)		Yes (n = 17)	No (n = 85)	
Corrected age	37 weeks [3]	38 weeks [3]	0.27	5.5 months [1.6]	4.9 months [1.1]	0.30
Lung compliance	0.84 [0.29]	1.28 [0.46]	< 0.001	1.53 [0.77]	1.94 [1.01]	0.12
Lung resistance	84.6 [30.7]	69.6 [29.9]	0.06	46.0 [19.0]	40.09 [17.5]	0.21
Tidal volume	6.1 [0.94]	7.2 [1.43]	< 0.01	6.9 [1.4]	7.3 [1.6]	0.42
Respiratory frequency	60.5 [10.8]	57.5 [12.5]	0.4	41 [10.3]	37.4 [9.0]	0.14
Weight (grams)	2518 [618]	2395 [751]	0.53	6180 [1121]	6423 [1166]	0.45
Length (centimeters)	45.8 [4.6]	45.7 [3.4]	0.92	62.3 [4.8]	63.5 [4.9]	0.41

Table 2 also shows the values for lung mechanics among children with and without bronchopulmonary dysplasia at the corrected age of 4-8 months. It can be seen that the premature children with bronchopulmonary dysplasia had mean compliance that was lower than that of children without bronchopulmonary dysplasia, and had higher resistance. However, there were no statistically significant differences between infants with and without bronchopulmonary dysplasia over time. 

**Figure 2:**
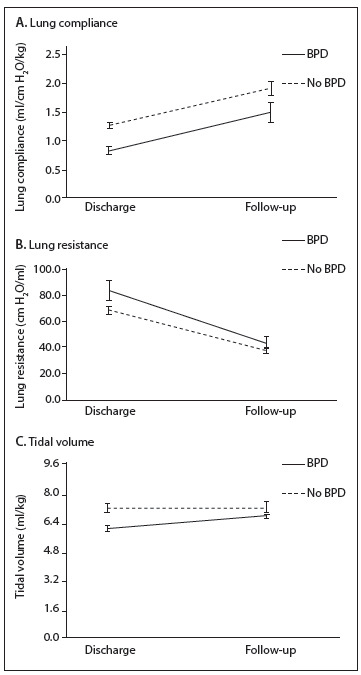
Lung mechanics in very low birth weight at discharge and at follow-up (4-8 months corrected age).

The increased in lung compliance was similar between the two groups as the children became older (Figure 2a). The lung resistance decreased over time for both groups. This decrease was greater among the children with bronchopulmonary dysplasia (Figure 2b), but the difference was not statistically significant. There was an increase in tidal volume among the children with bronchopulmonary dysplasia as their age increased, but the tidal volume among the children without bronchopulmonary dysplasia remained almost the same (Figure 2c). The increases in body weight and height/length were similar for the two groups over time.

## DISCUSSION

The most important result from this study was that the differences in lung function (compliance and tidal volume) that were observed at the time of hospital discharge were no longer observed at the follow-up assessment conducted at the age of 4-8 months.

This study showed that, at the time of medical discharge, the lung function damage among the premature infants with bronchopulmonary dysplasia was greater than that of the infants without the disease. Even considering the current lower severity of the bronchopulmonary dysplasia, the lung compliance was significantly abnormal in these children, given that it was 35% lower than in the children without the disease. Other studies have reported lung compliance values at the age of 2-4 months of age that were 30-50% lower than those of control children born at term. Moreover, these studies reported that the lung resistance in children with bronchopulmonary dysplasia was twice the value found in children in the control group.^14^,^15^

The anatomical and physiological formation of the lungs during the prenatal and postnatal periods depends on a complex relationship between factors that control vascular development and airway differentiation. Prematurity, exposure of lung epithelial and endothelial cells, high oxygen level, use of ventilation support, presence of infection and presence of patent ductus arteriosus may compromise the process of lung vascular and bronchoalveolar maturation, thus resulting in complications at an early stage of life (bronchopulmonary dysplasia) or later on.^16^ Hence, these adverse conditions may cause abnormalities in the lung function of prematurely born children and these may be intensified in children who develop bronchopulmonary dysplasia.^8^

Lower compliance results from the greater elastic limitation of the lungs of infants with bronchopulmonary dysplasia. Hjalmarson et al.^8^ reported that at the gestational age of 40 weeks, the specific compliance of the respiratory system of healthy premature infants born at gestational ages between 25 and 33 weeks was 73% of the compliance of children born at term. Although our study used a method for assessing lung compliance and not respiratory system compliance (which would make measurements of lung elastic recoil and thoracic wall recoil combined), the results show the same impairment as seen in other studies.^14^,^15^

Although there was a difference in the pulmonary function test parameters at the time of medical discharge, both groups reached lung compliance, resistance and volume values close to normality in the tests at 4-8 months. Although abnormal lung mechanics have been described in infants with bronchopulmonary dysplasia at the time of discharge from the neonatal intensive care unit,^4^ the longitudinal follow-up of lung mechanics in these patients is unclear.

The increases in lung and airway sizes over the first year of life may result in normalization of lung compliance and lung resistance.17 This probably contributed towards the sharp increase in tidal volume values seen in the children with bronchopulmonary dysplasia in the present study. These values were close to normality among the children without bronchopulmonary dysplasia at the time of medical discharge, and remained almost the same at 4-8 months. 

Smaller airway caliber may be a result of anatomical differences or subclinical inflammatory processes. Children with small airway caliber, reflected in impairment of lung function, may be at higher risk for wheezing during the first years of life. During childhood, a viral infection may induce reduction in the size of the peripheral airways, which will result in wheezing in children who already have a preexisting condition of small airway caliber,17 such as in the case of children with bronchopulmonary dysplasia. The results from the present study showed that lung function improved as the child grew older, but the compliance among children with bronchopulmonary dysplasia remained inferior to that of children without bronchopulmonary dysplasia and the resistance remained higher. This implies that even with growth, lung function seems to result from the prematurity and from any clinical complications that occurred soon after birth.18 This is concordant with the study by Gerhardt et al.,14 who reported that as children with bronchopulmonary dysplasia grew, their pulmonary compliance improved. However, it was still only 80-90% of the values seen in the controls at the ages of 2-3 years.

The present study shows the importance of assessing the pulmonary mechanics of very low birth weight preterm infants. However, there is a need for further studies in order to indicate which method would be best for assessing lung function during the various phases of the first year of life. This need can be seen from our sample, given that although the lung compliance values increased over time in the population of children with bronchopulmonary dysplasia, they still remained below those of the controls without the disease. 

It has also been shown in the literature that children born prematurely but without respiratory disease also have abnormal expiratory flows and that at least up to the age of two years, these flows continue to present values lower than those of the controls born at term.19 Baraldi and Filipone showed that abnormal flows continued up to adulthood.^20^

There is a need to carry out early analysis on several lung function parameters in order to identify these abnormalities earlier. It needs to be asked whether these infants with normal pulmonary compliance values at a corrected age of 4-8 months would also have normal values for forced expiratory flow and vital capacity at this age. There is a need for functional biomarkers for identifying children at risk of subsequent respiratory morbidity.^21 ^The population of preterm infants and carriers of bronchopulmonary dysplasia is more susceptible to respiratory morbidity, wheezing and development of asthma, according to the literature.^22^ Use of lung function tests among these children as a follow-up measure could contribute towards early clinical intervention and improvement of survival. 

Although the estimated sample size was 21, only 17 infants with bronchopulmonary dysplasia were enrolled in this study. This may be a limitation of the study, because this smaller number of children may have contributed towards lack of detection of any difference between the groups with regard to some features. Therefore, the present results should be interpreted with caution.

Another of the limitations of this study was the varying ages at which the second lung function test was performed. Because respiratory infections occur frequently among children and it is recommended to wait for a minimum of three weeks thereafter before the test is taken, this meant that we had to include a corrected age range from 4 to 8 months in the examination protocol. Another constraint was the high number of children excluded from the study because of respiratory infections presented at the time of the second test. This is a population susceptible to respiratory morbidity, which hinders availability of the entire population for the longitudinal tests. 

One of the strengths of this study was that it enabled sequential evaluation over the course of the first year of life, using a less invasive technique to document the lung function of very low birth weight preterm infants. However, a single evaluation of lung function at a follow-up after 4-8 months can be considered to be a limitation of the study.

There are few studies reporting serial measurements of lung function during childhood, thus showing the difficulties in making these measurements in this age group.^17^,^23^ In Brazil, few studies on pulmonary mechanics close to the time of medical discharge and over the course of the first year of life of very low birth weight premature children have been conducted.24,25 Thus, the present study provides an important contribution in this field of knowledge. 

## CONCLUSION

Differences in lung mechanics were observed between infants with and without bronchopulmonary dysplasia at the time of hospital discharge, but these differences were no longer detected at a corrected gestational age of 4 to 8 months. The lung mechanics of all the infants improved over this period of time.
